# No Question about Exciting Questions in Cell Biology

**DOI:** 10.1371/journal.pbio.1001734

**Published:** 2013-12-10

**Authors:** Thomas D. Pollard

**Affiliations:** 1 Department of Molecular, Cellular and Developmental Biology, Yale University, New Haven, Connecticut, United States of America; 2 Department of Molecular Biophysics and Biochemistry, Yale University, New Haven, Connecticut, United States of America; 3 Department of Cell Biology, Yale University, New Haven, Connecticut, United States of America

## Abstract

Veteran cell biologist Tom Pollard offers his view that the future of this field lies in quantitative mechanistic studies to explain how biological systems work.

## Summary

Although we have a good grasp of many important processes in cell biology, including knowledge of many molecules involved and how they interact with each other, we still do not understand most of the dynamical features that are the essence of living systems. Fortunately, we now have the ability to dissect biological systems in enough detail to understand their dynamics, including the use of mathematical models to account for past observations and predict future experiments. This deep level of mechanistic understanding should be our goal—not simply to satisfy our scientific curiosity, but also to understand the causes of disease well enough to predict risks, make early diagnoses, and treat effectively. Many big questions remain to be answered before we reach this goal of understanding cellular dynamics.

## Introduction

Professor Philipp Jolly famously advised Max Planck in 1878 not to enter the field of physics, because “almost everything is already discovered, and all that remains is to fill a few unimportant holes” [1]. Of course, physics has since gone from one breathtaking discovery to another. Just now, physicists are trying to figure out dark matter and dark energy, about which little is known except that they account for most of the matter and energy in the universe.

The history of science shows that the search for fundamental knowledge about nature unfolds steadily over centuries, always expanding the frontiers and never reaching what one would call an end point. Fields may get stuck in a rut and have to reinvent themselves from time to time with a paradigm shift [2], but so far no field of science familiar to me has run out of fundamental questions, all arising from previous work. Yet some serious scientists think that the spectacular progress in cellular and molecular biology since 1950 has already provided answers to most of the big questions in cell biology. The rest of us find that their predictions of the end of our discipline are way off the mark. How is this difference of opinion possible?

I suggest that these divergent points of view arise from two complementary visions about what is interesting and important in biology. Some focus on “what happens?” An example might be “molecular motors derive energy from ATP hydrolysis to power molecular movements.” Other scientists want to know “how does it work?” To use the same example, they want to understand how molecular motors convert the hydrolysis of ATP into force and how this fundamental reaction explains muscle contraction and cytokinesis.

I am firmly in the “how does it work?” camp, but many successful biologists are more interested in “what happens?” Think about your own loyalties. Once, I asked Francis Crick about his orientation and was not surprised to learn that he was more interested in “what happens.” This fascination with “what happens” led him away from molecular biology into neuroscience and the quest to understand consciousness.

By the turn of this century we understood a lot about “what happens,” so we had answers to many big questions in cell biology. The core principles of cell biology were defined in the 1950s and 1960s (Box 1), and our knowledge of “what happens” on the molecular level has improved dramatically since then. In my own field, all of the molecular motors and proteins that regulate the dynamics of actin filaments and microtubules were discovered over the past 40 years. By 2002, the first edition of the Pollard and Earnshaw cell biology textbook [3] accurately associated many of these motors and regulatory proteins with particular cellular movements. Indeed, all of cell biology expanded our grasp of the molecular basis of life during a golden age in the 1980s and 1990s, revealing much about “what happens” and answering many important questions.

**Box 1. Big Ideas in Cell Biology**

(Adapted from Pollard and Earnshaw, 2007 [3])

1. Genetic information stored in one-dimensional chemical sequences in DNA (occasionally RNA) is duplicated and passed on to daughter cells.
2. One-dimensional chemical sequences stored in DNA code for both the linear sequences and three-dimensional structures of RNAs and proteins and ultimately the architecture of cells and tissues.
3. Macromolecular structures assemble from subunits.
4. Membranes separate cells from their external environment, form biochemically distinct compartments in eukaryotic cells, and grow by expansion of preexisting membranes.
5. Signal–receptor interactions target cellular constituents to their correct locations.
6. Many cellular constituents move by diffusion, but energy-consuming pumps and motors move some constituents and whole cells.
7. Receptors and signaling mechanisms allow cells to adapt to environmental conditions.
8. Molecular feedback mechanisms control molecular composition, growth, and differentiation.

Nevertheless, this knowledge about molecules and their interactions does not come close to explaining how any biological system works. For example, simply knowing the protein components of the nuclear pore cannot explain transport between the nucleus and cytoplasm. Similarly, a deletion or depletion experiment showing that one of these proteins is required for transport does not explain the transport process. If this level of analysis were adequate, we would already know the mechanisms of most diseases, and drug companies would have lots of validated drug targets rather than being chronically short of them.

What's still missing in many areas of cell biology is an understanding of how molecules form the dynamical systems that bring the cell to life. Understanding dynamical processes is impossible from a list of their parts and their connections. Thus, many deep questions remain about the very essence of life, how life originated, and how cells and organisms have evolved.

## The Strategy to Understand Mechanisms

Fortunately, we have a proven strategy and the tools required to answer most any mechanistic question about dynamical systems of molecules in living cells and organisms. The strategy is reductionism with an emphasis on understanding how systems of molecules interact and respond to changes of conditions in the short term, and how organisms adapt on evolutionary time scales using similar molecules to come to diverse solutions. This strategy goes far beyond taking biological systems apart, however. It emphasizes the value of documenting the activities of molecules both in the test tube and in live cells, and it uses mathematical models and simulations to integrate information and test ideas. Independently, none of the elements of this strategy can explain how a system works at the cellular or organismic levels. Rather, each approach contributes to reach a synthetic understanding.

**Figure 1:**
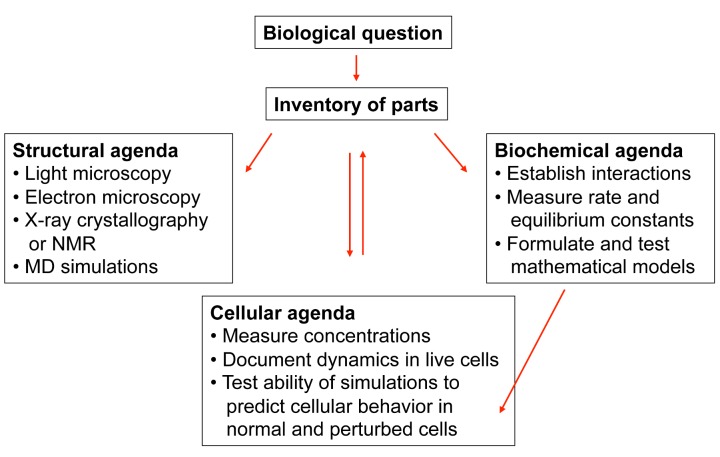
Diagram of the reductionist–synthetic strategy to characterize biological mechanisms. The boxes show the steps and research methods that contribute to understanding molecular mechanisms in cell biology. Arrows show the progression of the work starting with the definition of a biological question, followed by collecting an inventory of the relevant molecules and then three large areas of research: structural studies, cellular observations and biochemical characterization. When simulations of the hypothesis fail to account for observations in cells (bottom box), the investigator must reexamine their assumptions about the participating molecules (upward center arrow) and the participating reactions (right box) and the numerical parameters used in the simulations. Simulations and observations converge as understanding improves.

This reductionist–synthetic strategy for learning how things work (Figure 1) starts with framing a good question, one that is important and tractable. Virtually every aspect of cell biology still has profound questions to be answered. Just open any chapter of a cell biology textbook or attend a scientific meeting to find some examples. How did life on earth begin? How do polypeptides fold into stable proteins? How do non-coding RNAs contribute to cellular function? How do cells differentiate and self-organize into an organism? How do 100 billion neurons reliably establish their connections in the human brain using only information encoded in the genome? What limits regeneration of human tissues?

The second step is to identify the parts list for each biological process. One must catalog the participating molecules and link each to a process. Genomics, genetics, clinical medicine, comparative biology, and biochemistry all contribute to finding the molecules. Completing this inventory has been and will continue to be a major quest for cell biologists, since one cannot understand mechanism without a good inventory.

The third step in the strategy is to characterize the mechanisms of action of each participating molecule and to explain their participation in dynamical systems. Three lines of work, summarized below, provide complementary information about these mechanisms: structure, biochemistry and cellular dynamics. Typically, all three are implemented in parallel; however, proceeding from structure to biochemistry to cells can be most efficient, since prior information about structure informs the design of biochemical experiments, and knowledge of both strengthens the quality of the questions and experiments at the cellular level.

### The Structural Agenda

Discovery of fundamental information about structure at all levels will continue to be the bedrock of cell biology. Typically a combination of structural methods contributes to answering most mechanistic questions. X-ray crystallography has matured to the point where it is accessible to the biology community, so every lab with an interesting molecule should aspire to obtain its atomic structure. Expert crystallographers remain essential to improve methods and determine challenging structures of large macromolecular complexes. NMR can determine structures of molecules of modest size and provides unique information about their dynamics. Supercomputers have expanded the reach of molecular dynamics simulations, which will grow in importance in cell biology. Most importantly for cell biology, electron tomography and super-resolution fluorescence microscopy are providing ever more detailed views of cellular architecture with better spatial and temporal resolution.

### The Biochemical Agenda

The first steps are to characterize the properties of each molecule, including its interactions with partners. The use of classical biochemistry and genetics to establish networks of molecular interactions is now augmented by large-scale proteomics and genomics strategies that provide a broad sweep of interaction maps. A compelling test that all the parts have been found is reconstitution of a biological process from isolated components.

Understanding the dynamics of molecular systems depends on knowing the kinetic and thermodynamic parameters of the reactions. Most cell biologists are unfamiliar with methods to make these measurements, but they are actually relatively simple [4],[5]. Even with these parameters in hand, intuition fails when trying to understand biochemical pathways with more than two or three components, especially if the system involves feedback loops. Fortunately, one may formalize hypotheses in mathematical models and use computer simulations to test ideas and explore mechanisms. Powerful software is available for cell biologists to do this work without a high level of training in mathematics or computer science [5].

### The Cellular Agenda

Cell biologists have long tested their ideas by observing cells treated with drugs or carrying mutations that compromise specific molecules. (Note: the difference between the geneticist's view that a molecule is essential if it is required for viability, and the biophysicist's view that a molecule is essential if it is required for a process to run at the normal rate.) These experiments usually reveal if the compromised molecule participates in the process, but not how the system operates. That understanding requires information about the rates of reactions, which in turn depends on knowing the concentrations of the molecules of interest in cells, information rarely known by cell biologists. Fortunately, calibrated fluorescence microscopes with digital cameras can now measure global and local protein concentrations in live cells [6]–8, making the goal of calculating reaction rates in cells and subcellular compartments feasible.

Testing mechanistic hypotheses requires information about the dynamics of the molecules in live cells and how systems of molecules adapt to change. Measurements in live cells are essential to learn how the crowded environment in a cell influences reaction rates compared to typical biochemical measurements in dilute solutions. Historically, measurements have been done on samples of many cells but it is now appreciated that more can be learned from studying one cell at a time. Even genetically identical cells can behave distinctly, and this variability may be a vital part of the biology.

Several approaches are available to study dynamics in cells. Some processes can be seen visibly to proceed over time (think, for example, of endocytosis or the cell cycle), so one can document the time-course as events unfold. For other dynamic systems (transcription or protein secretion, for example), a pulse-chase approach or a response to perturbation (addition of stimulant or inhibitor) will show how the system adapts over time. Mutations are also useful for perturbing dynamics, particularly when the mutant gene product has been well-characterized biochemically.

## Integrating Cellular and Molecular Information to Test Mechanisms

One can test mechanistic hypotheses by asking if simulations of models can reproduce the observed dynamics in cells. In my experience, our hypotheses often fail this test, but we then make progress by letting these failures inform us about the shortcomings of the hypothesis. One refines the model by finding additional reactions and/or better parameters (arrows on the right in Figure 1), which usually leads toward a convergence between the simulations and observations, as illustrated by two papers from my lab [9],[10].

Cellular observations useful for testing mechanistic hypotheses require rigorous experimental design. For example, expressing fluorescent fusion proteins from plasmids at unknown levels in the presence of the wild-type protein has limited usefulness for quantitative experiments. New methods now allow cell biologists to express, at physiological levels, mutated genes for tagged proteins encoded directly in the genomes of animal cells and under control of the native promoter [11]—a proven approach in yeast cell biology. This not only allows for quantitative measurements but may also reveal that the tag compromises the function of the protein. Another example is using atomic structures to design proteins or RNAs lacking one or more specific structural domains rather than truncating or otherwise modifying them based on the arbitrary criterion of residue number.

Improved methods have always driven progress and will continue to do so in the future. New concepts will emerge naturally from research based on a good strategy to delve deeply into any question. The main limitations facing experimental biology today, I believe, are neither technical nor conceptual. Unfortunately, financial resources are limiting the ability of biologists to implement the reductionist–synthetic strategy to its most useful extent.

## The Long View

Cell biologists have enough fundamental, mechanistic questions to maintain the strength of the field for decades more. Some scientists may view mechanistic studies in cell biology as only adding detail to an essentially completed picture. I urge them to appreciate that mechanistic work *is* the future of cell biology, especially its practical applications. This quest is no less fundamental than discovering the nature of dark matter and dark energy rather than simply knowing that they must exist.
